# Identification and characterization of *CsYP* in regulating chloroplast development and cucumber peel color

**DOI:** 10.1093/hr/uhag043

**Published:** 2026-03-02

**Authors:** Yuanyuan Cui, Sen Li, Haoying Wu, Xi Zhao, Xiao Zhang, Yuming Dong, Yaru Wang, Menghang An, Lin Yang, Xiaofeng Chen, Yiqun Weng, Huazhong Ren, Xingwang Liu

**Affiliations:** College of Horticulture, China Agricultural University, Beijing 100193, China; College of Horticulture, China Agricultural University, Beijing 100193, China; College of Horticulture, China Agricultural University, Beijing 100193, China; College of Horticulture, China Agricultural University, Beijing 100193, China; College of Horticulture, China Agricultural University, Beijing 100193, China; College of Horticulture, China Agricultural University, Beijing 100193, China; Sanya Institute of China Agricultural University, China Agricultural University, Sanya, Hainan 572000, China; College of Horticulture, China Agricultural University, Beijing 100193, China; College of Horticulture, China Agricultural University, Beijing 100193, China; College of Ocean and Agricultural Engineering, Yantai Institute of China Agricultural University, Yantai, Shandong 264670, China; USDA-ARS, Vegetable Crops Research Unit, Horticulture Department, University of Wisconsin, 1575 Linden Dr., Madison, WI 53706, USA; College of Horticulture, China Agricultural University, Beijing 100193, China; Sanya Institute of China Agricultural University, China Agricultural University, Sanya, Hainan 572000, China; College of Horticulture, China Agricultural University, Beijing 100193, China; Sanya Institute of China Agricultural University, China Agricultural University, Sanya, Hainan 572000, China

## Abstract

Skin color is a crucial quality trait in cucumber fruit, yet the regulatory mechanisms underlying cucumber skin color remain poorly understood. In this study, we characterized a cucumber natural mutant displaying yellow peel, and identified a key gene *yellow peel* (*CsYP*) through map-based cloning. *CsYP* encodes a rhodanese-like protein with a Rhod domain. A single-base insertion results in premature termination of protein translation, leading to the yellowing pericarp phenotype in the natural mutant. To further investigate the function of CsYP, two knockout lines, *yp-1* and *yp-2*, were generated using CRISPR-Cas9 technology. Phenotypic investigation of *yp-1* and *yp-2* revealed a significant yellowing of the pericarp starting from 6 days after pollination, consistent with the natural mutant phenotype. Additionally, our study revealed an interaction between CsYP and Cscytb6f, a cytochrome b6-f complex iron–sulfur subunit, suggesting a collaborative role of CsYP and iron–sulfur proteins in regulating cucumber peel color. These findings provide novel insights into the regulatory mechanisms underlying cucumber peel color and broaden our understanding of this important trait.

## Introduction

Cucumber (*Cucumis sativus* L.) is an important vegetable crop that belongs to Cucurbitaceae. It is extensively cultivated worldwide and plays a vital role in human diet. The immature fruit of the cucumber is commonly used in its fresh or pickled form [1–3]. The skin color of cucumber is a crucial factor that influences consumer acceptance and marketability. In northern China markets, for instance, dark green cucumber skins are more popular than light green ones, making it a key objective in cucumber breeding. Overall, cucumber is a valuable crop that provides essential nutrients for human nutrition and holds significant economic value in the market [4].

There is a wide range of color variations observed in immature cucumber fruits, ranging from dark green to striped, yellow green, white, and light green. In recent years, genetic studies have focused on investigating the skin color characteristics of cucumber fruits. Previous research primarily focused on genetic analysis and gene mapping, leading to the identification of six major genes that control cucumber skin color, namely *lgp*/*CsARC5* [5], *w*/*CsAPRR2* [6], *lgf*/*CsYcf54* [7], *ygp*/*CsMYB36* [8], and *D*/*CsDULL* [9]. Despite identifying and locating the genes related to cucumber peel color, the molecular mechanism underlying their regulation is still poorly understood. Recent studies have suggested that the known cucumber skin color genes *CsARC5* [5], *w* [6], *CsYcf54* [7], and *CsMYB36* [8] function similarly to plant leaf color genes, potentially influencing chloroplast development or participating in key steps of chlorophyll synthesis [10–12]. Furthermore, some research implies that skin color changes are associated with the degree of skin lignification and the abundance of certain pigmented flavonoids (particularly anthocyanins and chalcones), which provides new insights into the causes of skin color variations [13, 14]. Although studies on the regulatory mechanisms of chlorophyll synthesis in plants have gained relative clarity, it is necessary to explore novel mechanisms underlying the regulation of cucumber peel color.

Chloroplast development plays an essential role in fruit coloration. To fully understand the molecular mechanisms behind chloroplast development, extensive research is needed. Additionally, other co-factors, such as the iron–sulfur (Fe–S) cluster, can also impact fruit coloration and nutrition [15]. Fe–S clusters play crucial roles in vital cellular processes, including enzyme catalysis, electron transfer, and the regulation of gene expression, repair, and translation of the protein [16–19]. In *Arabidopsis thaliana*, point mutations in the *SufB* gene, which is involved in Fe–S cluster biogenesis, lead to chlorophyll degradation and accumulation of demagnetic chlorophyllic acid A. *SufB* mutants in tobacco (*Nicotiana tabacum*) exhibit a reduced number of chloroplasts [20–23]. By screening and identifying cofactors involved in chloroplast development, we may gain insights into the changes in peel color during fruit ripening.

Chloroplasts house numerous proteins that are essential for iron–sulfur clusters. The proper assembly of iron–sulfur clusters is crucial for chloroplast biogenesis and the realization of their key biological functions. While the biosynthesis of iron–sulfur clusters has been extensively studied in bacteria and yeast [24], research regarding the biological origin of Fe–S clusters in higher plants is lacking [24–26]. Rhodanese (Rhod), an enzyme widely distributed in plants and other organisms [27]. It plays a role in providing sulfur atoms necessary for the construction of chloroplast proteins via Fe–S clusters [28]. This initial reaction is crucial for the formation and development of chloroplasts. However, there are limited reports on related research in crops.

In our study, we have successfully identified a candidate gene, *CsYP*, that encodes a Rhod and controls peel color in cucumber, through gene mapping and whole genome sequencing. Subsequent RNA-seq analysis confirmed significant changes in the expression levels of genes involved in photosynthetic pathways and chloroplast development in the *yp* mutant. Furthermore, protein interaction assays demonstrated a direct interaction between CsYP and Cscytb6f, a subunit encoding the cytochrome protein complex. These findings provide valuable insights into the molecular mechanism of how Rhod affects peel color in cucumber. This innovative research sheds light on the understanding of pericarp color and opens up new avenues for further investigations in this field.

## Results

### Yellow peel mutants in cucumber are often associated with abnormalities in chloroplast development

**Figure 1:**
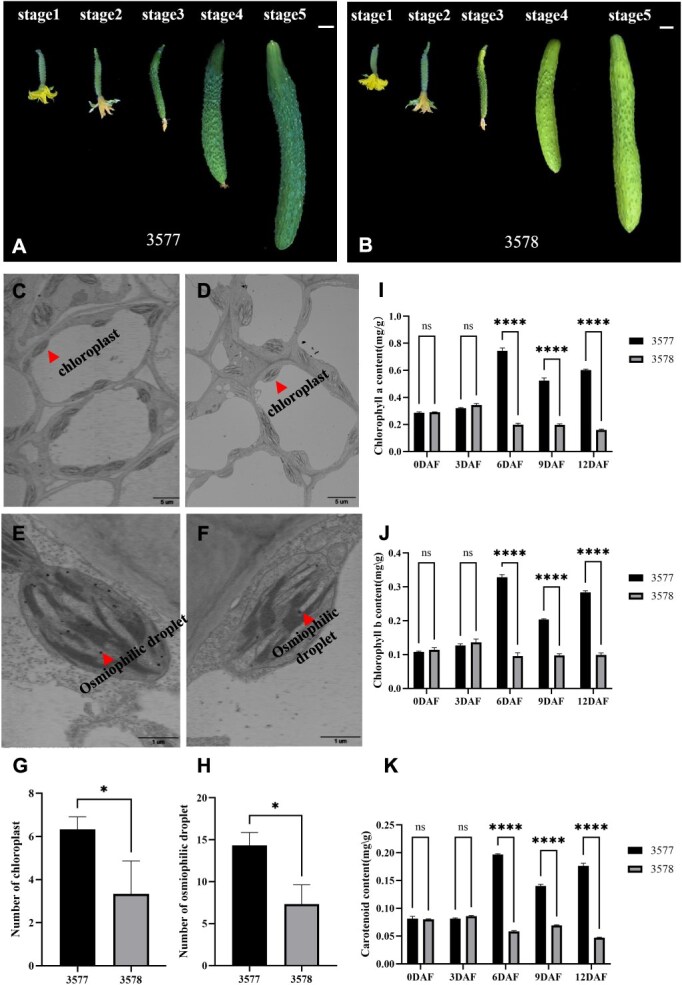
Phenotypic characterization of *yp* mutants. (A) Phenotypic characteristics of wild type (3577) fruits at different developmental stages. (B) Phenotypic characteristics of mutant (3578) fruits at different developmental stages. Stages 1–5 represent 0, 3, 6, 9, and 12 days after flowering, respectively. DAF is short for days after flowering. (C and D) Transmission electron microscopy of chloroplast at 5000× in wild-type pericarp (C) and in mutant (D). (E and F) Transmission electron microscopy of chloroplast at 30 000× in wild-type pericarp (E) and in mutant (F). (G) Quantification of chloroplast count in wild type and mutant within the same field of view. (H) Quantification of Osmiophilic drop numbers in wild type and mutant within the same field of view. (I, J, K) Pigment content analysis in pericarp of wild type and mutant at different stages, including Chlorophyll a (I), Chlorophyll b (J), and Carotenoid (K). Scale bar = 1 cm (A, B). Error bars represent SD from 3 biological reps. ^*^ indicates *P* < 0.05. ^****^ indicates *P <* 0.0001.

In this study, we obtained a pair of near-isogenic line materials, namely 3577 (wild type) and 3578 (mutant), through hybridization at the early stage. To examine the peel color differences between wild-type and mutant fruits, we divided the fruit development into five stages. Notably, the mutant fruits exhibited distinct skin color variations, specifically showing yellowing starting from the third stage (~6 days after pollination). As the fruits continued to develop, the phenotype of fruit yellowing became more pronounced during the fourth and fifth stages (Fig. 1A and B).

Previous research has indeed indicated that the development of abnormal chloroplasts could result in yellow-peel mutants [9]. In our study, we aimed to investigate the underlying cause of fruit yellowing in the specific mutant. To do so, we examined the chloroplast morphology during the key stage of peel color change using transmission electron microscopy. At a magnification of 5000×, we observed a significant decrease of 47.4% in the number of chloroplasts in the mutant compared to the wild type (Fig. 1C, D, and  G). Additionally, at a magnification of 30 000×, we found that the chloroplasts in the mutant exhibited shrinkage and incomplete development, with a reduction of 48.8% in the number of osmiophilic droplets compared to the wild type (Fig. 1E, F, and  H). The biochemical essence of osmiophilic droplets is plastoglobules, which are spherical lipid droplets enclosed by a half-unit membrane in the chloroplast stroma. Their biological significance lies in storing substantial carbon and chemical energy without increasing osmotic pressure or volume, for use during darkness, nighttime, or periods of high energy demand, such as seed germination and damage repair. These results suggest that the yellow peel phenotype is associated with a decrease in the number of chloroplasts and malformation of chloroplasts.

To gain further insight into the causes of yellow peel, we measured the changes in pigment content in pericarp at different stages of fruit development. There was no significant difference in pigment content in pericarp between the wild type and the mutant in the first and second stages. However, starting from the third stage, the distinction became apparent. For the detailed analysis in the third stage, the mutant exhibited a substantial decrease, including a 72.6% reduction in chlorophyll a (Fig. 1I), a 75.1% reduction in chlorophyll b (Fig. 1J), and a 71.1% reduction in carotenoids (Fig. 1K), compared to the wild type. These results indicate that the yellow peel phenotype may be attributed to a reduction in pericarp pigment concentration. Overall, the abnormal development of chloroplast appears to be the primary cause of yellow peel in this mutant.

### Inheritance analysis reveals recessive single-gene control of yellow peel trait in cucumber

We investigated the inheritance mode of yellow peel segregating populations (F_1_, F_2_, BC_1_P_1_, and BC_1_P_2_) from crosses between 3461 (P_1_) and 3578 (P_2_). 3461 of these were cucumber materials with a typical green peel, while 3578 with a typical yellow peel. Materials with distant relations were chosen to build the mapping population in order to simplify mapping. In the F_1_ generation, we observed 20 green peel plants and no yellow peel plant, indicating a segregation ratio of 1:0. However, in the F_2_ generation, we observed a 3:1 segregation ratio with 269 green peel plants and 95 yellow peel plants. Furthermore, the BC_1_P_1_ generation exhibited a 1:0 segregation ratio, while the BC_1_P_2_ generation displayed a 1:1 segregation ratio (Table 1). These findings suggest that yellow peel is a recessive characteristic governed by a single gene.

**Table 1:** Genetic analysis of different generations

**Lines/populations**	**Total**	**Green**	**Yellow**	**Green:Yellow**	***P* (*χ*** ^**2**^ **test)**
3461 (P_1_)	20	20	0	20:0	
3578 (P_2_)	22	0	22	0:22	
F_1_	20	20	0	1:0	
F_2_	364	269	95	3:1	0.24
BC_1_P_1_	56	56	0	1:0	
BC_1_P_2_	56	29	27	1:1	0.08

### Genetic mapping and identification of candidate genes associated with yellow peel trait

To determine the genetic localization of yellow peel genes, we selected 60 green pericarp plants and 60 yellow pericarp plants from the F_2_ generation for BSA sequencing. The parents of these plants were also re-sequenced. We used SNP-index and ΔSNP index to analyze the BSA data to pinpoint the key regions related to the target traits.

**Figure 2:**
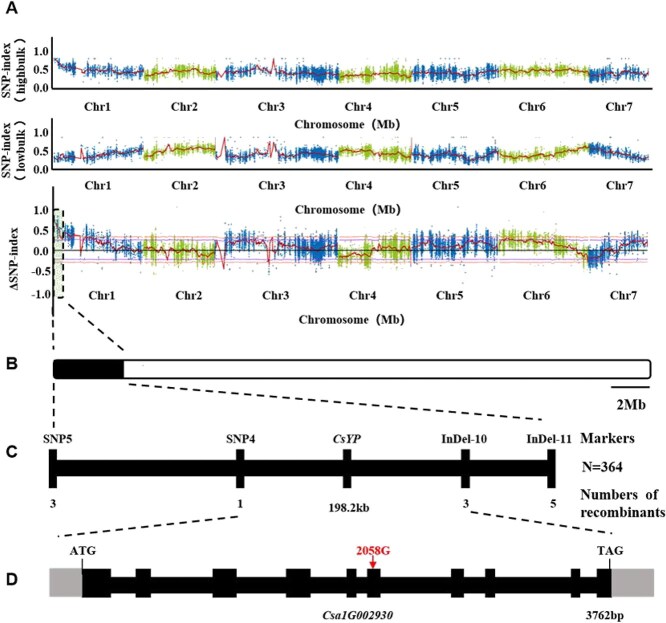
Fine mapping of *CsYP.* (A) ΔSNP index plot showing the difference in SNP index between green peel and yellow peel. ΔSNP index, SNP index (green peel) − SNP index (yellow peel). The orange dashed line represents the threshold of ΔSNP index. The colored dots represent the calculated ΔSNP index value of each SNP locus and red lines represent the fitted ΔSNP index. (B) Rough positioning result of *CsYP* based on the ΔSNP index plot. (C) Distribution of InDel and SNP markers in the rough mapping interval. (D) Gene structure and sequence variation of candidate gene *CsYP* (*Csa1G002930*). The red arrow indicates the site of a guanine (G) base insertion.

The ΔSNP index, a parameter indicating the frequency differences of single nucleotide genotypes, provided a comprehensive reflection of the SNP variances within the different mixed pools. The analysis revealed that a region affecting yellow peel was localized at 0 to 3 Mb of chromosome 1 (Fig. 2A).

Subsequently, InDel and SNP markers were designed within the BSA localization region to further refine the mapping. Through this approach, we successfully obtained 8 recombinant single strains from a population of 90 recessive individuals. The yellow peel gene was ultimately mapped between the molecular markers InDel-10 and SNP-4 on chromosome 1, spanning a physical distance of 198.2 kb (Fig. 2B and C). Upon examining the whole genome annotation of cucumber, we identified a candidate interval containing 42 genes (Table S1).

### CsYP, encoding a rhodanese, identified as a candidate gene regulating yellow peel trait

Through analysis of Bulked Segregant Analysis (BSA) sequencing data from the parental lines 3461 (green peel) and 3578 (yellow peel), we identified 21 mutations across 42 genes within the candidate region (Table S2). To further explore the genetic basis of the yellow peel phenotype, we compared the mutation profiles of 3578 (yellow peel) and 3577 (green peel). Notably, a single InDel was identified at position 554242 on chromosome 1, present in both sequenced parents—3461 (green peel) and 3578 (yellow peel)—as well as in wild type 3577 (green peel). This InDel occurs in the sixth exon of the gene *Csa1G002930*, introducing a frameshift mutation that leads to premature termination of translation (Fig. 2D).

To identify candidate genes associated with cucumber yellow peel, we performed RNA-seq on wild type and mutant peel tissues at Stage 3. By analyzing the transcriptome data, we found differential expression in 7 out of 42 genes in the candidate regions. Among these, 3 genes were up-regulated, with the most significant up-regulation observed in *Csa1G004180*, exhibiting an expression level of 3.35 times higher than that of the wild type. Additionally, four genes were down-regulated, with *Csa1G002930* showing the most significant decrease in expression, with a reduction of 78% (Table S3). Based on the sequence mutation of yellow peel materials and the RNA-seq results of candidate genes, *Csa1G002930* was selected and designated as *yellow peel* (*CsYP*) for further characterization.

### Knockout of *CsYP* confirms its role in yellow peel phenotype

**Figure 3:**
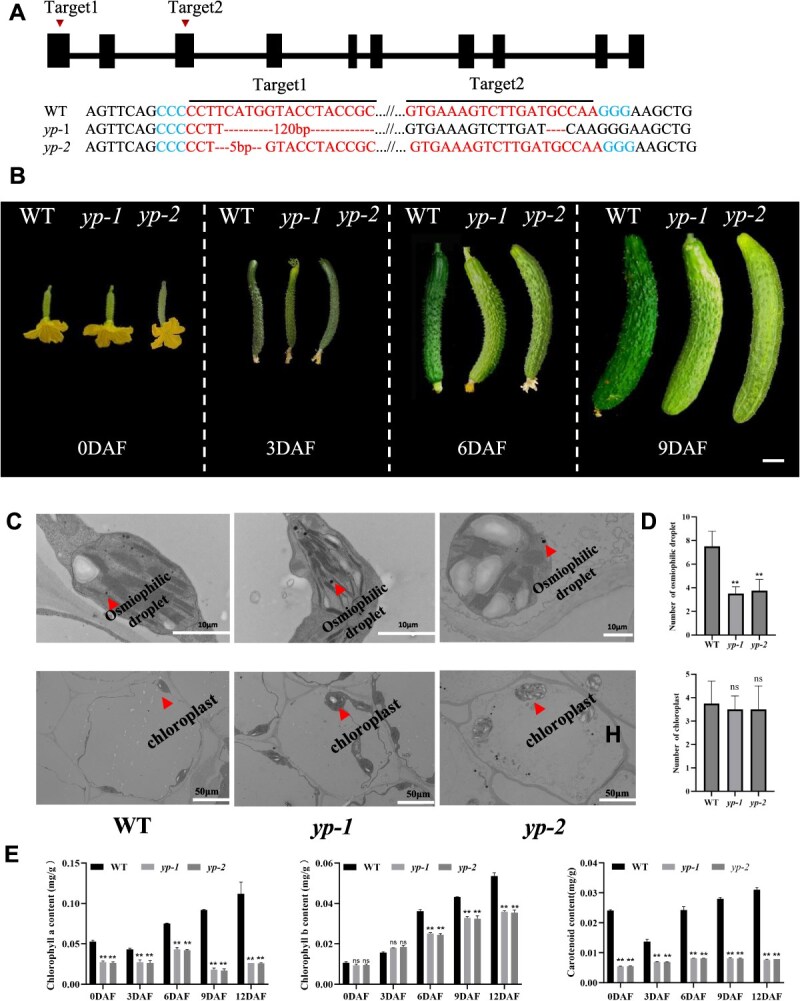
Phenotypic characterization of *CsYP* CRISPR/Cas9 transgenic plants. (A) Variation observed in *CsYP* knockout transgenic lines (*yp-1* and *yp-2*). The start sequence is at the left most end, and the black square denotes the exon. (B) Comparison of phenotypes between *yp-1*, *yp-2,* and wild-type (3667). (C) Transmission electron microscopy observation. (D) Quantification of chloroplast count and quantification of osmophilic drop numbers in wild type and *yp-1* and *yp-2* mutant within the same field of view. (E) Determination of pigment in leaf green of *yp-1*and *yp-2* mutant and wild-type. Scale bar = 2 cm (B). Error bars represent SD from 3 biological reps. ^**^indicates *P < *0.01.

To investigate the functional role of *CsYP* in controlling cucumber peel yellowing, we employed CRISPR/Cas9 to create an independent transgenic line *yp-1* and *yp-2* in the 3667 background (green peel) (Fig. 3A; Fig. S1). Interestingly, the transgenic plants exhibited a phenotype of peel yellowing, which manifested at 6 days after pollination, consistent with the mutant phenotype observed in fruit color at 0, 3, 6, 9 days after pollination (Figs 1B and 3B). Leaf yellowing was observed in transgenic plants at the seedling stage, but this symptom diminished significantly as development progressed. Meanwhile, the difference in pericarp color between the mutant and the wild type progressively increased. Given that this study focuses on fruit color changes, systematic investigations were subsequently conducted exclusively on fruit coloration (Fig. S2). Further analysis of chlorophyll a, chlorophyll b, and carotenoid levels revealed a significant reduction in the transgenic lines, comparable to that observed in the yellow mutant (Fig. 3E). Transmission electron microscope demonstrated chloroplast abnormalities as well as a reduction in the amount of osmiophilic droplets in the peels of *yp-1* and *yp-2* (Fig. 3C and D). Collectively, these results strongly support the notion that *CsYP* is a key regulatory gene of cucumber peel yellowing by regulating chloroplast development and pigment content.

### Analysis of *CsYP*, a putative candidate gene for yellow peel regulation

**Figure 4:**
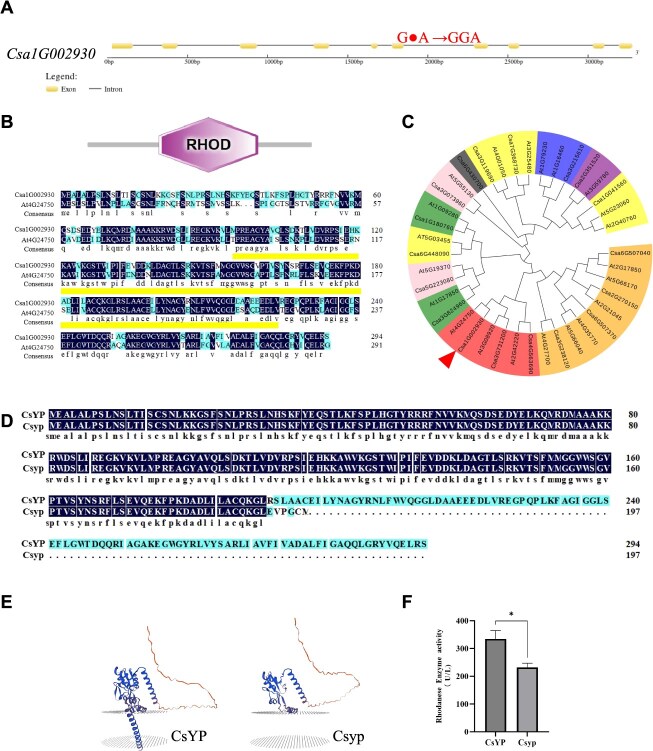
Analysis of *CsYP.* (A) Schematic diagram of the gene structure and mutation site in CsYP. (B) CsYP gene structure domain and the sequence conservation analysis. The underlined region is the Rhod domain. (C) CsYP phylogenetic tree analysis. (D) Alignment of the protein sequences from CsYP and Csyp. (E) CsYP and Csyp protein structure prediction (SWISS—MODEL, http://swissmodel.expasy.org). (F) Rhodanese enzyme activity determination in CsYP and Csyp. Error bars represent SD from 3 biological reps. ^*^indicates *P* < 0.05.

Through gene cloning in both green peel and yellow peel parents, we identified a single-base G insertion in the sixth exon of the yellow peel materials. This insertion eventually led to premature termination of translation and the production of an abnormal target protein (Fig. 4A and D).

To better understand the genetic and functional differences of *CsYP* between cucumber and other species, we analyzed CsYP homologous proteins. Phylogenetic analysis revealed that CsYP had the highest homology with At4G24750 in *Arabidopsis* (Fig. 4C). A sequence conservation analysis revealed that this gene is highly conserved with its homolog in Arabidopsis thaliana, and both possess the Rhod domain, which is typical for thiocyanate enzymes (Fig. 4B). Notably, At4G24750 is annotated as a Rhod/cell cycle-associated phosphatase superfamily protein, which is located in chloroplasts. Previous reports have shown that *CsYP* and *At4G24750* co-distributed within the branches located in chloroplasts, suggesting their potential involvement in regulating chloroplast development [29]. Prediction and analysis of the protein structure revealed that the mutant protein exhibits a markedly altered conformation compared to the wild type (Fig. 4E). Furthermore, a significant decrease in enzyme activity was observed (Fig. 4F). This finding suggests that *CsYP* may regulate yellow peel by influencing chloroplast development.

### Temporal and spatial expression patterns of *CsYP*

**Figure 5:**
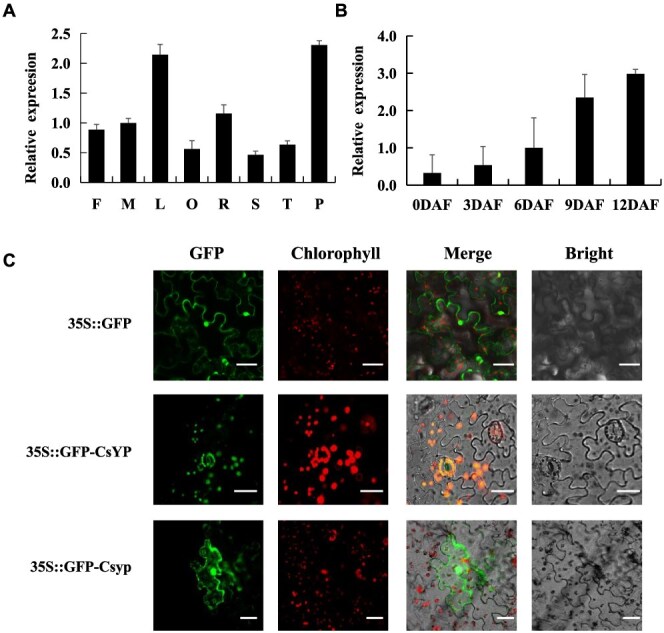
Temporal and spatial expression analysis of *CsYP.* (A) *CsYP* gene expression in various parts of cucumber. F, female flower; M, male flower; L, leaf; O, ovary; R, root; S, stem; T, tendril; P, ovary peel. (B) Quantitative real-time PCR of *CsYP* at different stages of fruit development. 0 DAF, the flowering day; 3 DAF, the 3 days after flowering; 6 DAF, the 6 days after flowering; 9 DAF, the 9 days after flowering; 12 DAF, the 12 days after flowering. (C) CsYP subcellular localization. 35S::GFP is the control group, 35S::GFP-CsYP is the expression vectors of unmutated gene 35S::GFP-Csyp is the expression vectors of mutated gene. Scale bar = 50 μm.

To explore the expression patterns of *CsYP*, we conducted qRT-PCR analysis on samples from different tissue parts. Our results revealed variable expression levels of *CsYP* across different tissues, with the highest expression in the pericarp, indicating its involvement in the regulation of peel development (Fig. 5A). To further elucidate the expression trend of *CsYP* during different stages of fruit development, we performed quantitative Real-time PCR, which demonstrated an increase in *CsYP* expression corresponding to the degree of peel yellowing. This finding highlights the pivotal role of *CsYP* as a key regulatory factor of peel color (Fig. 5B). Subcellular localization confirmed the presence of CsYP in chloroplasts, and an evident change in localization was observed in the mutant, providing further evidence of the gene's crucial function in peel yellowing process (Fig. 5C).

### CsYP interacts with Cscytb6f

**Figure 6:**
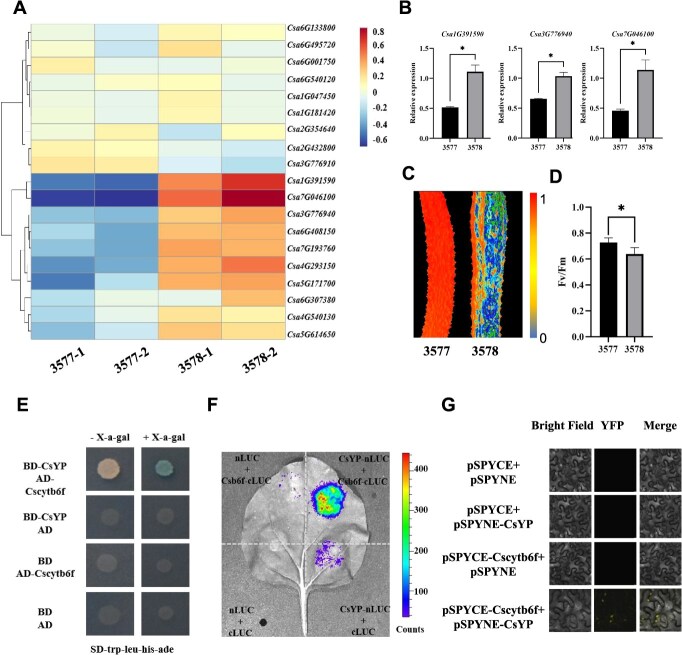
RNA-seq and protein interaction in CsYP and Cscytb6f. (A) Heat maps of 3577 and 3578 iron–sulfur cluster related differential genes (color bar on the right indicates normalized fold changes). (B) qRT-PCR validation of three DEGs from RNA-seq with peel samples of 3577 and 3578. Error bars represent the mean ± standard deviation (*n* = 3). The asterisks indicate a significant difference (*P* < 0.05; *t*-test). (C) False-color fluorescence image, color-coded according to the scale (0 to 1) displayed on the right. (D) Fv/Fm. (E) Yeast two-hybrid results of CsYP and Cscytb6f. (F) Luciferase complementation imaging (LCI) assay of the interaction between CsYP and Cscytb6f. (G) BiFC assay showing the interaction between Cscytb6f-YFPC and CsYP-YFPN in *Nicotiana benthamiana* leaves.

To gain insights into the mechanisms underlying CsYP-mediated pericarp yellowing, we used RNA-seq data to identify potential interacting factors with CsYP (Fig. 6A). The findings of the screening revealed significant alterations in the expression levels of *Csa1G391590*, *Csa3G776940*, and *Csa7G046100* in the two materials (Fig. 6B–D). *Csa7G46100* exhibited the most distinct changes in transcriptional expression, suggesting its potential involvement in the *CsYP*-mediated regulation of peel color.

This gene showed the highest homology with *AT4G03280* in *Arabidopsis*, which encodes the Rieske Fe–S core of the cytochrome b6f complex. Studies have indicated that mutations in *AT4G03280* lead to bleaching phenotype, yellow leaves, blocked photosynthetic electron transport, and lack of photoautotroph. Furthermore, the predicted location of this gene in Arabidopsis is within the chloroplast. The interaction between CsYP and Cscytb6f was further verified using Y2H (Fig. 6E), luciferase complementation assay (Fig. 6F), and Bimolecular fluorescence complementation (BiFC) (Fig. 6G). To further evaluate the photosynthetic efficiency, we measured the Fv/Fm ratio in both wild-type and mutant Stage 3 fruits. The results revealed that the Fv/Fm value was significantly lower in the mutant compared to the wild type (Fig. 6C and D). These results demonstrate the interaction between CsYP and Cscytb6f, suggesting a collaborative role of CsYP and Fe–S proteins in regulating cucumber peel color.

## Discussion

### *CsYP* is a novel gene resource for regulating pericarp yellowing in cucumber

Through gene mapping and RNA-seq, we identified *CsYP* (*Csa1G002930*) as a candidate gene involved in the regulation of yellow peel in cucumber. Comparative analysis of known yellowing genes did not reveal any corresponding mutations in our mutants, suggesting that CsYP represents a novel gene resource for controlling the yellowing phenotype.

Among the previously reported cucumber skin color genes, such as *CsARC5*, *CsYcf54*, *CsIST*, and *CsMYB36,* their function in chloroplast formation or crucial stages of chlorophyll synthesis influencing peel color change are yet to be confirmed [30]. In contrast, our study demonstrated that the knockout of *CsYP* in the *yp-1* and *yp-2* lines resulted in a significant reduction in pigment content and deformities in chloroplast structure, along with a reduced number of osmiophilic droplets. In conclusion, CsYP plays a functional role in regulating pericarp yellowing.

### As rhodanese-like protein, CsYP may cooperate with iron–sulfur proteins to participate in chloroplast metabolism

*CsYP (Csa1G002930)* is annotated as a Rhod-like protein in the cucumber genome (Chinese long v2)*.* Rhod enzymes catalyze the transfer of sulfur from donor to sulfur acceptor substrates. Comparative genomic analysis has revealed that the STR family consists of multiple genes clustered into different groups, with some members located in chloroplasts [31]. *CsYP* belonged to the fifth STR family together with three other genes in this cluster (*AT1G17850*, *AT4G24750*, and *AT2G42220*), implying the involvement of this family in chloroplast-related pathways. Among them, *CsYP* shares the highest homology with the Rhod/cell cycle associated phosphatase superfamily *AT4G24750*. *In vitro* experiments have shown that Rhod activity is associated with the Rhod domain, which catalyzes the formation of thiocyanates and sulfites. The catalytic site is usually located in plastids. The specific functions of many members of this superfamily are still unknown, but proposed roles include cyanide detoxification [32, 33], sulfur transfer for iron–sulfur cluster biosynthesis or repair [34, 35]. Notably, CsYP interacts with Cscytb6f, a gene encoding cytochrome b6-f complex iron–sulfur subunit. This interaction suggests that CsYP may be involved in chloroplast metabolism in coordination with iron–sulfur protein, providing new insights into the functional mechanism of STR family genes. Our results suggest a potential link between peel color and iron and sulfur metabolism, thereby offering an applicable approach to enhance peel color by modulating the nutrient status of iron and sulfur.

### The role of iron–sulfur cluster-related genes in chloroplast development and pericarp color deserves attention

Fe–S cluster proteins are essential for chloroplast synthesis. These clusters serve as essential cofactors in organisms and are necessary for chloroplast biogenesis and vital biological processes. While the biosynthesis of Fe–S clusters has been extensively studied in bacteria and yeasts, there is still much to uncover regarding their biogenesis in higher plants. In this study, we found that CsYP interacts with Cscytb6f, which encodes a cytochrome complex Fe–S protein. In previous research on *Arabidopsis*, mutation of this gene resulted in a leaf bleaching phenotype, suggesting that abnormal Fe–S protein may cause a chlorotic phenotype [36, 37] .

Fe–S clusters are evolutionarily conserved, multifunctional prosthetic groups that coordinate with proteins through covalent interactions to mediate essential biochemical processes, including electron transfer, enzymatic catalysis, and metabolic regulation. In chloroplasts, iron–sulfur clusters have a significant impact on photosynthesis. The newly discovered and cloned gene *CsYP* has been found to possess the ability to influence the production and repair of Fe–S clusters. Our Y2H results showed that CsYP interacts with the cytochrome complex iron–sulfur protein Cscytb6f (Fig. 6E and F). This further suggests that CsYP may affect the color change of pericarp by regulating iron–sulfur protein. Transcriptome analysis identified numerous changes in the transcription levels of Fe–S cluster-related genes, suggesting their involvement in the development of yellow peel. By introducing the regulation mechanism of peel color into the Fe–S pathway, this study provides a novel understanding of the mechanism underlying peel color regulation.

**Figure 7:**
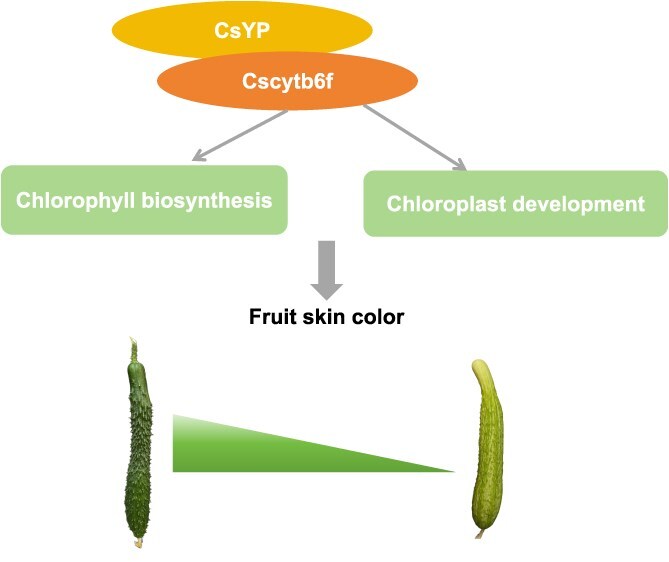
A proposed model for the regulatory mechanism of *CsYP* in controlling fruit skin color.

In this study, we successfully identified the key gene *CsYP* responsible for regulating pericarp yellowing through gene mapping, and its functional verification confirmed its role in pericarp color change. Moreover, through RNA-seq data, Y2H and LCI, we found that Cscytb6f interacts with CsYP. Previous studies have shown that Fe–S protein is essential for chloroplast development [38, 39]. Therefore, we speculate that CsYP may influence the formation of iron–sulfur clusters in chloroplasts through its interaction with Cscytb6f, thereby affecting chloroplast development and ultimately explaining the causes of peel yellowing from a more microscopic perspective (Fig. 7). In conclusion, our study provides an innovative insight into the regulation mechanism of cucumber peel color by incorporating the iron and sulfur synthesis pathway.

## Materials and methods

### Plant materials and phenotype of yellow peel

In this study, a pair of near-isogenic line (NILs) materials 3577 and 3578 created in our laboratory were used for the analysis of phenotype and transcriptome. 3578 (yellow peel) is a natural mutant of 3577 (green peel), and there are significant differences in pericarp color. Inheritance analysis and gene mapping were conducted on 3461 (green peel) and 3578 (yellow peel). More concretely, 3577, 3578, and 3461 is an inbred line selected from a high-generation inbred line developed by our laboratory, respectively. After seeding, cucumber seedlings complete the growth process in a solar greenhouse.

### Group construction

In the spring of 2018, a group of 20 F_1_ individuals was generated through the cross between parental lines 3461 (P_1_) and 3578 (P_2_). In the autumn of the same year, the F_1_ individuals were subjected to self-pollination to obtain a population of 364 F_2_ individuals. Additionally, the F_1_ individuals were backcrossed with both parental lines 3461 (P_1_) and 3578 (P_2_) to generate separate populations of 56 BC_1_P_1_ individuals and 58 BC_1_P_2_ individuals. In the spring of 2019, all the parental lines (P_1_ and P_2_), F_1_ individuals, F_2_ population, BC_1_P_1_ individuals, and BC_1_P_2_ individuals were planted in Qing County, Hebei Province (38° 37′N, 116° 49′E) for population investigation and genetic analysis.

### Pigment content determination

To measure pigment content, samples were obtained from the wild-type and mutant fruits at 3-day intervals from the day of pollination to 12 days after pollination. The procedure involved dissolving 0.2 g of fruit material in a 95% ethanol solution, make up to 25 ml, incubating it in the dark for 24 hours at 25°C. Using 95% alcohol as the blank control and using a UV spectrophotometer at wavelengths of 645 nm and 470 nm. Measure the absorbance value and calculate it. The formula is as follows:

Chlorophyll a concentration (mg/l) =13.95 × A665–6.88 × A649.

Chlorophyll b concentration (mg/l) =24.96 × A649–7.32 × A665.

The concentration of carotenoids (mg/l) = (1000 × A470–2.05 × Ca-114.8 × Cb) /245.

Pigment content (mg/g) =0.025 L × C/W.

l is a unit of liquid volume (liter); C represents the pigment concentration (mg/l); W represents the fresh weight of the sample (g); V is the final volume of the extract solution (ml). Three biological replicates were set up and then statistics were conducted.

### Transmission electron microscope

To examine the peel tissues at the stage of noticeable yellowing (Stage 4), 3 × 3 mm squares were excised from the peel and immediately placed in a pre-cooled fixing solution and transported in ice boxes to maintain their integrity. Slices were prepared following fixation, dehydration, encapsulation, solidification, sectioning, and staining. The prepared samples were subsequently observed using a transmission electron microscopy to analyze the cellular structures in detail. Model HT7800 is the particular transmission electron microscope.

### Fine mapping of *CsYP*

For the fine mapping of CsYP, a total of 60 plants with either green or yellow peel, derived from 3461 and 3578 materials, were selected for Bulked Segregant Analysis (BSA). The resequencing was performed at Shanghai Ouyi. The cucumber reference genome was utilized for raw read processing, genome-wide SNP frequency, and SNP analysis using BWA and GATK software.

To achieve fine localization, Insertion/Deletion (InDel) and derived Cleaved Amplified Polymorphic Sequences (dCAPs) primers were designed following the method described by Zhai *et al*. [9]. These specific primers were then used to amplify and detect the target gene in the F_2_ plants. As a result, the *CsYP* gene was successfully mapped to a specific region of 198.2 kilobases on chromosome 1.

### Subcellular localization

For subcellular localization analysis of CsYP, total RNA was extracted from plant samples using the Huayueyang RNA extraction kit and the Tiangen inversion kit was used to generate complementary DNA (cDNA). In brief, the full-length coding sequence of CsYP without the stop codon was cloned from cDNA of 3577 and inserted in the 35S::GFP expression vector (owned by our laboratory) to form a CsYP–GFP fusion construct. The resulting vector was transformed into the 3577 and 3578 plant materials using Agrobacterium GV3101 strain for transformation. To observe the localization of the gene, infected tobacco samples were examined using confocal microscopy at an excitation wavelength of 640 nm. For detailed experimental procedures, refer to the methodology described by Nie *et al*. [40]. Information on primers used in this experiment is provided in Table S4.

### qRT-PCR analysis

qRT-PCR analysis was performed on 3577 main tissue parts and pericarp tissues at different stages of fruit development. UBI/UBQ genes were used as reference genes. Three biological replicates and technical replicates were set for each treatment, and the data were analyzed using the 2^−ΔΔCT^ method [41]. Reactions were carried out using three biological and three technical replicates for each sample. Information on primers used in this experiment is provided in Table S4.

### Determination of Rhodanese activity

The enzyme activities of CsYP and Csyp were detected using a Rhod ELISA kit.

This kit adopts a double-antibody one-step sandwich enzyme-linked immunosorbent assay (ELISA). First, the specimen, standard, and HRP-labeled detection antibody are successively added to the pre-coated micro-wells with Rhod antibody. After incubation and thorough washing, the substrate TMB is used for color development. TMB is converted into a blue color under the catalysis of peroxidase and then into a final yellow color under the action of acid. The intensity of the color is positively correlated with the Rhod content in the sample. The absorbance (OD value) is measured at a wavelength of 450 nm using a microplate reader, and the activity of the sample is calculated accordingly.

### RNA-seq analysis

Peel tissues from 3577 and 3578 were used for RNA-seq analysis. Three biological replicates of each treatment were sent on dry ice, and the RNA-seq experiment was conducted by Shenggong Biotechnology Corporation (https://www.sangon.com/). The RNA-seq data were collected using the Illumina platform, and Trimmomatic software conducted quality preprocessing on the raw data [42]. Clean reads and the Chinese long V2 cucumber data base (http://cucurbitgenomics.org/organism/2) were used for sequence alignment and further investigation of genetic variants.

### Knockout of *CsYP*

To knockout CsYP, a CsYP-CRISPR vector was constructed. The targets for CsYP were designed using the CRISPR-p website, and two pKSE402 vectors were used as carriers for the construction. The constructed CsYP-CRISPR vector was verified through sequencing to confirm its successful construction. For genetic transformation, the CsYP-CRISPR vector was used to transform the Changchunmici (3667) variety. The regenerated plants were screened by GFP fluorescence and sequencing of the *CsYP* gene.

The detailed methods are as follows.

After soaking the cucumber seeds in warm water, they were rinsed under running water for 4 to 5 hours. Then, they were disinfected with 70% alcohol on a super-clean workbench for 1 minute. Finally, they were rinsed five to six times with sterile water and inoculated onto the MS basic medium. After the cucumber seedlings without bacteria have grown for about 5 to 6 days and the two cotyledons have not fully opened, the growth points are cut off. The upper half of both cotyledons is cut off by 1/2 to 2/3, and the hypocotyl is longitudinally cut, with a 2-mm hypocotyl retained, and then placed on the cotyledonous bud induction medium for 2 days. Take the pre-cultured cotyledon segments, infect them with activated Agrobacterium bacterial solution (with 100 μmol/l AS added to the solution) for 15 minutes, aspirate the surface liquid on sterile absorbent paper, cultivate them in the co-culture medium for 2 days, and then place them in the screening medium. The regenerated plants were screened by GFP fluorescence and sequencing of the *CsYP* gene.

### Yeast two-hybrid assay

The CDS sequences of *CsYP* and *Cscytb6f* were ligated into the pGADT7 and pGBKT7 vectors using homologous recombination method. The plasmid was extracted and transformed into yeast strain AH109. The successful transformation of the vectors was confirmed by growth on selective medium lacking tryptophan and leucine (-Trp/-Leu, DDO) for 3 days at 28°C. To verify the interaction between CsYP and Cscytb6f, yeast colonies from the previous step were further tested on selective medium lacking leucine, tryptophan, histidine, and adenine (-Leu/-Trp/-His/-Ade, QDO) supplemented with X-α-Gal.

### Luciferase complementation assay

The CDS sequence of *CsYP* was ligated into pCambia1300-nLUC vector using homologous recombination method, and the CDS sequence of *Cscytb6f* was ligated to pCambia1300-cLUC vector. The plasmid was extracted and transformed into Agrobacterium GV3101.

Tobacco leaves at 4 weeks of seedling age were divided into 4 equal parts. Each part of the leaf was injected with a combination of bacterial solution on the dorsal side of the leaf. The injected tobacco was kept in darkness for 2 days and the back side of the leaf was sprayed with a fluorescent substrate. The material was processed for 5 minutes in dark condition, and subsequently, the fluorescence was observed.

### Bimolecular fluorescence complementation

The full-length or segmented cDNA sequences of CsYP and Cscytb6f were cloned into pSPYNE or pSPYCE vectors to construct fusions expressing YFP^N^ and YFP^C^. The fusion expression vectors were transformed into Agrobacterium tumefaciens and the mixture of two different plasmids of equal volume was transformed instantaneously into Tobacco leaves. The injected Tobacco leaves plants were cultured for 3 days at 28°C for 8-hour light/16-hourdarkness conditions, and the fluorescence of green fluorescent protein (GFP) was observed in the area of Tobacco leaves injected with Agrobacterium using a confocal microscope.

### Chlorophyll fluorescence parameter determination

The maximum quantum yield of photosystem II (Fv/Fm) and other key photosynthetic parameters were measured using a chlorophyll fluorescence imaging system (IMAGING-PAM-MAXI, Walz Heinz GmbH, Effeltrich, Germany). The measurements were performed on dark-adapted leaves (30 minutes), and the parameters were calculated with the accompanying ImagingWin v2.56zg software [43].

## Supplementary Material

Web_Material_uhag043

## Data Availability

The data supporting this article are accessible within the article itself and the online supplementary material.
