# Supplementary material for: Identification and characterization of CsYP in regulating chloroplast development and cucumber peel color
Source: Hortic Res. 2026 Mar 2;13(5):uhag043. doi: 10.1093/hr/uhag043 (PMC13156030; doi:10.1093/hr/uhag043)
Supplement: Web_Material_uhag043 [file web_material_uhag043.zip › Supplemental Tables.docx]

**Supplementary Data Table S1. Candidate genes annotation in the candidate interval**

| No | Gene | Range | Notation |
| --- | --- | --- | --- |
| 1 | Csa1G002790 | 495815..497198 | Anthranilate N-benzoyltransferase |
| 2 | Csa1G002800 | 497568..499103 | Protein SCARECROW |
| 3 | Csa1G002810 | 501847..502845 | Peptide methionine sulfoxide reductase MsrA |
| 4 | Csa1G002820 | 502969..505746 | Putative WD-repeat protein |
| 5 | Csa1G002830 | 506451..509905 | Abhydrolase domain-containing protein FAM108C1 |
| 6 | Csa1G002840 | 517062..518160 | WD repeat-containing protein |
| 7 | Csa1G002850 | 518756..520101 | Centromere protein X |
| 8 | Csa1G002860 | 520583..522860 | Receptor-like protein kinase |
| 9 | Csa1G002870 | 526662..529358 | Sieve element occlusion c |
| 10 | Csa1G002880 | 531138..534254 | Protein-L-isoaspartate O-methyltransferase |
| 11 | Csa1G002890 | 534041..536360 | RNA-binding protein |
| 12 | Csa1G002900 | 537068..541237 | Alkaline alpha galactosidase |
| 13 | Csa1G002910 | 544419..546125 | Glyoxal oxidase-related protein |
| 14 | Csa1G002920 | 546702..549784 | Copine, putative |
| 15 | Csa1G002930 | 552186..555947 | Rhodanese-like family protein-like protein |
| 16 | Csa1G002940 | 564885..565597 | Octicosapeptide/Phox/Bem1p domain-containing protein kinase |
| 17 | Csa1G002950 | 565892..566441 | Unknown protein |
| 18 | Csa1G003450 | 571184..574637 | Leucine-rich repeat-containing protein, putative |
| 19 | Csa1G003460 | 575753..577319 | MATE efflux family protein |
| 20 | Csa1G003470 | 577763..578995 | Multidrug resistance protein MdtK |
| 21 | Csa1G003480 | 580627..580998 | Unknown protein |
| 22 | Csa1G003490 | 581925..585370 | Multidrug resistance protein MdtK |
| 23 | Csa1G003500 | 586303..586775 | Unknown protein |
| 24 | Csa1G003510 | 588876..592262 | Protein kinase |
| 25 | Csa1G003520 | 592299..594662 | Pentatricopeptide repeat-containing protein |
| 26 | Csa1G003530 | 595079..597553 | Pentatricopeptide repeat-containing protein |
| 27 | Csa1G003540 | 606147..607595 | Elongation factor Tu |
| 28 | Csa1G004040 | 612649..613677 | Cytochrome P450, putative |
| 29 | Csa1G004050 | 615237..617749 | Putative receptor kinase |
| 30 | Csa1G004060 | 618042..620222 | U-box domain-containing protein |
| 31 | Csa1G004070 | 620814..621020 | Unknown protein |
| 32 | Csa1G004080 | 621120..624349 | ATP-dependent RNA helicase-like protein |
| 33 | Csa1G004090 | 624562..625167 | ATP-dependent RNA helicase dbp2 |
| 34 | Csa1G004100 | 634874..641524 | Amine oxidase |
| 35 | Csa1G004110 | 645459..645836 | Unknown protein |
| 36 | Csa1G004120 | 646345..646832 | MYB transcription factor |
| 37 | Csa1G004130 | 652611..655662 | Histone deacetylase |
| 38 | Csa1G004140 | 656458..658107 | Pentatricopeptide repeat-containing protein |
| 39 | Csa1G004150 | 658180..659035 | Unknown protein |
| 40 | Csa1G004160 | 659158..659958 | Putative uncharacterized protein |
| 41 | Csa1G004170 | 668136..670232 | LOB domain-containing protein |
| 42 | Csa1G004180 | 678390..679993 | Chromosome undetermined scaffold_34 |

**Supplementary Data Table S2. Variation sites within the candidate interval**

| No | Position | 3461 | 3577 | 3578 |
| --- | --- | --- | --- | --- |
| 1 | 494385 | T | C | C |
| 2 | 511240 | G | T | T |
| 3 | 554242 | T | T | TG |
| 4 | 689194 | T | G | G |
| 5 | 689819 | C | A | A |
| 6 | 690060 | G | C | C |
| 7 | 690405 | AT | A | A |
| 8 | 690952 | G | C | C |
| 9 | 690954 | A | C | C |
| 10 | 691355 | G | GA | GA |
| 11 | 691370 | A | AGAAAAAG | AGAAAAAG |
| 12 | 691612 | A | G | G |
| 13 | 691700 | G | A | A |
| 14 | 691709 | T | A | A |
| 15 | 691722 | A | G | G |
| 16 | 691909 | T | C | C |
| 17 | 692058 | A | T | T |
| 18 | 692478 | A | G | G |
| 19 | 692484 | G | T | T |
| 20 | 692491 | G | A | A |
| 21 | 692590 | TGTTCGTATGTAGTTTTACCCAACACC | T | T |

**Supplementary Data Table S3. Analysis of transcription differences of candidate genes**

| Gene ID | Fold change | Trend | Notation |
| --- | --- | --- | --- |
| Csa1G002890 | 2.01 | Up | RNA-binding protein |
| Csa1G004060 | 2.13 | Up | U-box domain-containing protein |
| Csa1G004180 | 3.35 | Up | Chromosome undetermined scaffold |
| Csa1G002810 | 0.52 | Down | Peptide methionine sulfoxide reductase MsrA |
| Csa1G002940 | 0.54 | Down | Octicosapeptide/Phox/Bem1p domain-containing protein kinase |
| Csa1G002950 | 0.73 | Down | Unknown protein |
| Csa1G002930 | 0.78 | Down | Rhodanese-like family protein-like protein |

**Supplementary Data Table S4. List of primers for expression and localization analysis**

| Primer name | Primer sequence |
| --- | --- |
| CsYP-cds-F | ATGGAAGCTCTGGCGCTTCCTTCCC |
| CsYP-cds-R | TCAGTGAGACCGTAATTCCTGAACA |
| CDS-1300F | AAGCTTCTGCAGGGGCCCGGGATGGAAGCTCTGGCGCTTCCTT |
| CDS-1300R | CATGGTACCGGATCCACTAGTGTGAGACCGTAATTCCTGAACA |
| Mt-1300-F | GGACGAGCTGTACAAGGTCGACATGGAAGCTCTGGCGCTTCCTTC |
| Mt-1300-R | GCTCGGTACCGGATCCACTAGTTCAGTGAGACCGTAATTCCTGAA |
| 1300-seq-F | TACAGCAAGAACGGAATGCGCGTGA |
| 1300-seq-F | GCAGATGAACTTCAGGGTCAGCTTG |
| CsYP-qRT-F | CTTGCATGCCAAAAGGGGTT |
| CsYP-qRT-R | CAAACGAGCAGAGTAAACCA |
