# Supplementary material for: Identification and characterization of CsYP in regulating chloroplast development and cucumber peel color
Source: Hortic Res. 2026 Mar 2;13(5):uhag043. doi: 10.1093/hr/uhag043 (PMC13156030; doi:10.1093/hr/uhag043)
Supplement: Web_Material_uhag043 [file web_material_uhag043.zip › Supplementary Data Figure.docx]

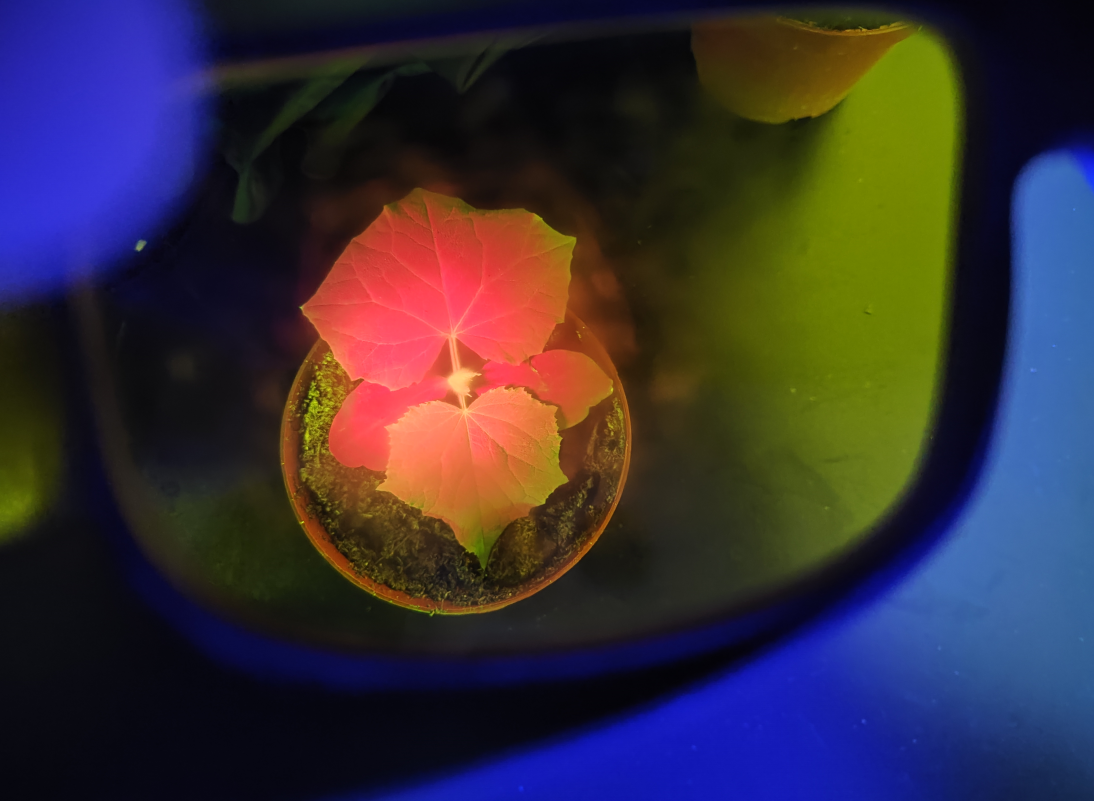

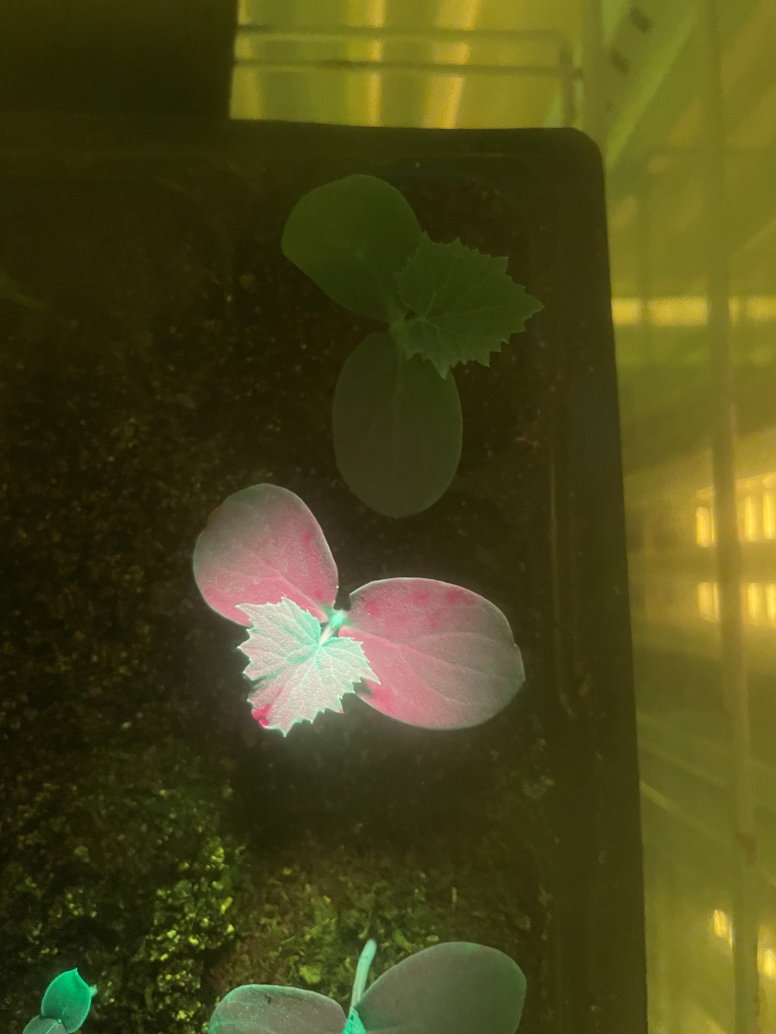


**A**

**B**

**Supplementary Data Figure S1. GFP fluorescence of transgenic lines**

(A) GFP fluorescence of transgenic lines observed in one leaf and one mind stage. (B) GFP fluorescence of transgenic lines observed in two leaves of one mind stage. Scale bar=2cm (A,B).


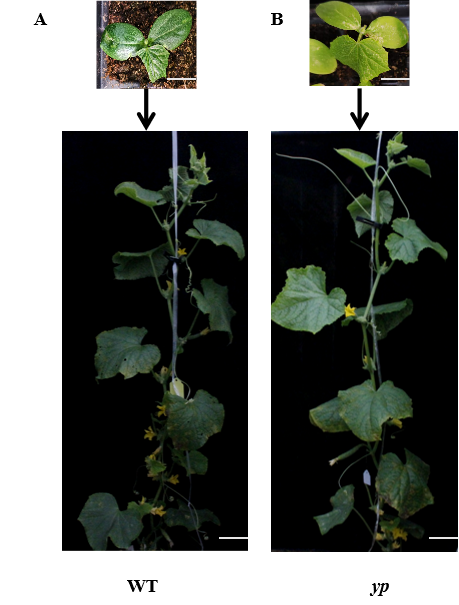


**Supplementary Data Figure S2. Phenotypes between transgenic lines and wild-type plants**

1. Color of wild-type leaves. (B) Color of *yp* leaves. Scale bar=4cm (A,B).
